# Report of Diseases, (No. 2,) from the 10th of December, 1822, to the 10th of March, 1823; Being a List of the Complaints Affecting Children, Treated at the Royal Infirmary for Sick Children

**Published:** 1823-04

**Authors:** John Webster

**Affiliations:** one of the Physicians to that Institution.


### STATISTICAL MEDICINE.

ART. I.—*Report of Diseases, (No. 2,) from the 10th of December, 1822, to the 10th of March, 1823; being a List of the Complaints affecting Children, treated at the Royal Infirmary for Sick Children.* By JOHN WEBSTER, M.D. one of the Physicians to that Institution.*

*Diseases affecting particular Organs.*

| | | | | |
|---|---|---|---|---|
| Head and Nervous System | | Hydrocephalus Acutus | 10 | |
| | | Epilepsia | 2 | |
| | | Paralysis | 1 | |
| | | Tetanus | 1 | = 14 |
| Organs of Respiration | | Hæmoptisis | 1 | |
| | | Tracheitis | 4 | |
| | | Bronchitis | 91 | |
| | | Pneumonia | 5 | |
| | | Pleuritis | 4 | |
| | | Phthisis | 1 | |
| | | Pertussis | 67 | =173 |
| Organs of Digestion | Mouth, &c. | Cynanche Parotidea | 1 | |
| | | ——— Tonsillaris | 3 | |
| | Stomach | Dyspepsia | 23 | |
| | Bowels | Constipatio | 6 | |
| | | Diarrhœa | 6 | |
| | | Dysenteria | 7 | |
| | Liver | Hepatitis Chronica | 1 | |
| | | Icterus | 1 | |
| | All three | Cholera | 18 | |
| | General | Marasmus | 3 | |
| | Worms | Lumbricus | 1 | |
| | | Ascarides | 12 | = 82 |
| Skin Eruptions | Acute | Variola Confluens | 3 | |
| | | Variola Discreta | 7 | |
| | | Varicella | 4 | |
| | | Morbilli | 12 | |
| | | Urticaria | 1 | |
| | Chronic | Impetigo | 1 | = 28 |

*Diseases affecting various Parts.*

| | | | |
|---|---|---|---|
| Fevers | Continua Simplex | 3 | |
| | ——— Cephalica | 13 | |
| | ——— Pleuritica | 4 | |
| | ——— Gastro-enteritica | 56 | |
| | Remittens | 19 | |
| | Intermittens, Tertiana | 2 | = 97 |
| Dropsies | Ascites | 1 | |
| Cachexia | | 3 | |
| Vaccinated | | 3 | = 7 |
| | Total number of patients | | 401 |

*Fatal Cases reported at the Infirmary:*—Bronchitis, 7; Variola Confluens, 3; Tracheitis, 2; Pneumonia, 2; Cholera, 2; Morbilli, 1; Pertussis, 1; Hydrocephalus, 1; Dyspepsia, 1; Lumbricus, 1; Marasmus, 1; Paralysis, 1.—Total, 23.

* We regret that Dr. WEBSTER's Report did not reach us till the present Number was nearly finished: on this account, we have been obliged to take the liberty of curtailing it very much.—EDITORS.

In the end of November, and early part of December, the most frequent disease was the gastro-enteritic variety of fever: during the hard frosty weather, about Christmas, it entirely disappeared, and inflammatory affections of the respiratory organs occupied its place. On the change to moist and rainy weather, which occurred in the early part of January, fever again became common; which a second time gave way to affections of the organs of respiration, on the appearance of the very severe storm about the middle of that month, at which period they were the chief complaints.

The plan recommended by the American physician, Dr. ARCHER, to vaccinate children when affected with pertussis, has been tried. Hitherto but few experiments have been made: consequently, the conclusion drawn may appear not sufficiently supported, when contrary to the more extensive experience of Dr. Archer. In three cases only, accurate results were obtained; of these, two were affected with hooping-cough of some standing at the time of vaccination: neither were in the least benefited by the operation; one, indeed, had more frequent returns of the disease after than before. The other child, who underwent vaccination with the usual object of protection from the small-pox, and at the same time had not the slightest symptom of hooping-cough, (nor was it then thought of,) became severely attacked with it about the seventh or eighth day, when the vaccine disease was exceedingly distinct: notwithstanding which, the hooping-cough was very frequent and obstinate, clearly showing the little influence cow-pox has upon pertussis. Although these facts are unfavourable, and appear conclusive, nevertheless other experiments shall be instituted when proper opportunities occur.

Of the twenty-three cases which proved fatal, seven were examined; of these I shall relate the appearances which presented themselves in one, a case of hooping-cough. The subject of it had remittent fever in November last; cholera in January, and never had enjoyed good health since, or for some time previously: it became affected with hooping-cough early in February, which was accompanied with great affection of the chest. It was a very violent case; and, though the hooping ceased three days previous to death, by the application of leeches to the forehead, the infant sunk from the great derangement of the organs of respiration. It is right to state, that hooping had been only observed for about ten days. On opening the cranium, the vessels on the surface, and in all the convolutions of the brain, were quite gorged with blood, and depositions of lymph were observed on various parts of both hemispheres, with adhesions of the anterior lobes to each other, which it required some force to separate; the choroid plexus was very vascular, and there was upwards of an ounce of water in the ventricles. The left lung adhered firmly to the pleura costalis; and at this point a large abscess was met with in its substance, full of pus, with the bronchial tubes opening into it. Several tubercles in various parts of the lungs, of both sides, were observed. The bronchia appeared to have been inflamed; and two small red spots were discovered on the internal surface of the larynx. The trachea was quite sound. The liver was much larger than ordinary, of a tuberculated form, and of a very white colour. The spleen was small, and had a very large patch of lymph, of a firm consistence, adhering to its superior edge.

